# Physiological stress and post-release mortality of white marlin (*Kajikia albida*) caught in the United States recreational fishery

**DOI:** 10.1093/conphys/cov066

**Published:** 2016-02-10

**Authors:** Lela S. Schlenker, Robert J. Latour, Richard W. Brill, John E. Graves

**Affiliations:** 1 Virginia Institute of Marine Science, College of William & Mary, PO Box 1346, Gloucester Point, VA 23062, USA; 2 National Marine Fisheries Service, Northeast Fisheries Science Center, James J. Howard Marine Sciences Laboratory, 74 Magruder Road, Sandy Hook, Highlands, NJ 07732, USA

**Keywords:** Air exposure, catch and release, handling practices, post-release mortality, stress physiology, white marlin

## Abstract

White marlin, a highly migratory pelagic marine fish, support important commercial and recreational fisheries throughout their range in the tropical and subtropical Atlantic Ocean. More than 10 000 individuals can be caught annually in the United States recreational fishery, of which the vast majority are captured on circle hooks and released alive. The probability of post-release mortality of white marlin released from circle hooks has been documented to be <0.02, but the associated physiological stress resulting from capture and handling techniques has not been characterized despite its importance for understanding the health of released fish. We examined the physiological response of 68 white marlin caught on circle hooks in the recreational fishery and followed the fate of 22 of these fish with pop-up satellite archival tags programmed to release after 30 days. Measures of plasma sodium, chloride, glucose and lactate concentrations taken from fish that were briefly and consistently (mean = 120 s, standard deviation = 40 s) removed from the water increased with angling time, but post-release mortality was inversely related to angling time. The probability of post-release mortality was predicted by elevated plasma potassium concentrations and was more than 10 times greater than has been previously reported for white marlin caught on circle hooks that were not removed from the water. This disparity in estimates of post-release mortality suggests that removal of fish from the water for physiological sampling greatly heightens stress, disrupts homeostasis and thus increases the probability of post-release mortality. Our results demonstrate that elevated concentrations of plasma potassium predict mortality in white marlin and that the probability of post-release mortality is highly dependent on post-capture handling procedures.

## Abstract (precis)

Understanding the physiological response of White Marlin, a popular sportfish, to catch-and-release fishing will aid management and conservation. White Marlin showed increased stress with angling time, but mortality was inversely related to angling time. Mortality was predicted by increased plasma potassium and may be heightened by air exposure.

## Introduction

Understanding the magnitude of post-release mortality is crucial for stock assessments, the development of effective fishery management plans and resource conservation (Campana *et al*., 2006). In the USA, >50% of recreationally caught marine fishes are released alive, but these individuals are most likely to experience some level of physiological stress and physical harm (Bartholomew and Bohnsack, 2005; Cooke *et al*., 2012). Understanding how angling practices affect stress, injury and the ability of an individual to regain homeostasis is crucial to reducing post-release mortality. Estimating probabilities of post-release mortality in large pelagic fishes (e.g. tunas and billfishes) has been hindered by the difficultly in working with these species in the field. However, deriving reliable estimates is especially crucial given the ecological significance of tunas and billfishes as highly migratory top predators that support important fisheries worldwide (Juan-Jordá *et al.*, 2011).

White marlin (*Kajikia albida*) have undergone >50% reduction in biomass over the last half-century throughout their range in the tropical and subtropical waters of the Atlantic Ocean (Juan-Jordá *et al.*, 2011), and the population is considered very unlikely to rebuild in the next 10 years [International Commission for the Conservation of Atlantic Tunas (ICCAT), 2012]. Despite these low biomass estimates, stock assessment models may still be overestimating white marlin population size owing to the misreporting of significant numbers of the morphologically similar roundscale spearfish (*Tetrapterus georgii*) as white marlin (Shivji *et al.*, 2006; Beerkircher *et al.*, 2009). White marlin are caught as bycatch by commercial fishing gear targeting tunas and swordfish (ICCAT, 2012), harvested as a food fish by artisanal fisheries (Arocha and Bárrios, 2009) and targeted in a multimillion-dollar recreational fishery along the US Atlantic coast and in other areas throughout their range (Skomal, 2007).

Billfish management regulations adopted over the last decade and increased angler conservation awareness have resulted in the release of the vast majority of white marlin captured by the recreational fishery (Horodysky and Graves, 2005). In recent years, the recreational fishery operating within the US mid-Atlantic region has captured and released ∼10 000 white marlin annually (Graves and Horodysky, 2015). Assuming an average size of 22 kg (Graves and Horodysky, 2015), the weight of white marlin released in that fishery is almost half of the total reported landings (∼400 metric tons) for the species (ICCAT, 2012). Accurate estimation of the probabilities of post-release mortality of white marlin captured by the US recreational fishery is therefore especially important given both the magnitude of this fishery and the low biomass of the population.

Pop-up satellite archival tags (PSATs) can provide information on post-release mortality coupled with detailed behavioural data. However, relatively few studies have used these devices to estimate specific probabilities of post-release mortality in pelagic fishes (Holland *et al.*, 1990b; Graves *et al.*, 2002; Musyl *et al.*, 2015). Previous research using PSATs has shown that post-release mortality of white marlin in the US recreational fishery is reduced significantly by the use of circle hooks (Horodysky and Graves, 2005; Graves and Horodysky, 2008). The effects of other variables (e.g. angling time and handling condition) have not been evaluated in the recreational fishery targeting white marlin. Obtaining statistical power for such estimates requires large sample sizes (Goodyear, 2002) that are cost-prohibitive because of the relatively high cost of PSATs (∼$4000 per tag; Horodysky and Graves, 2005; Kerstetter and Graves, 2008; Musyl *et al.*, 2011). There is currently a need for a cost-effective way to estimate probabilities of post-release mortality with robust sample sizes and improved statistical power. Physiological samples can be collected at low cost from a large number of fish and may provide information about the fate of fish stressed from the angling event, boatside handling, de-hooking and release procedures. Using PSATs in combination with physiological data can allow for a relatively low-cost, statistically powerful and empirically based method to infer post-release mortality in fishes (e.g. Moyes *et al.*, 2006; Marshall *et al.*, 2012).

Researchers examining the physiological response to exhaustive exercise (e.g. Iwama *et al.*, 2006) have analysed blood samples taken from fish in the field (Moyes *et al.*, 2006; Heberer *et al.*, 2010; Danylchuk *et al.*, 2014) and laboratory (Mazeaud *et al.*, 1977; Cicia *et al.*, 2012) to quantify levels of capture stress for species that are frequently released by recreational and commercial fisheries. The response to capture and handling typically initiates a suite of reactions that include the following: an accumulation of lactate in white muscle, a decrease in intracellular and extracellular pH, internal fluid shifts, splenic contractions releasing red blood cells into the general circulation, and red blood cell swelling, the latter two driving elevations in haematocrit (Wood, 1991; Kieffer *et al.*, 1995; Brill *et al*., 2008; Skomal and Mandelman, 2012). These biochemical and physiological responses can be measured and used to quantify the degree of physiological stress. However, this assumes the acquisition of a minimally stressed reference point or spectrum of known responses with which ‘stressed’ values can be compared.

Physiological disturbances resulting from exhaustive exercise may be of great enough severity to cause death (Wood *et al.*, 1983), but the identification of physiological predictors of post-release mortality has remained elusive. The stress response has been shown to be species specific and to be dependent upon the duration and nature of the stressor, and studies often observe few post-release mortalities, which limit statistical inferences regarding a specific cause(s) of death (Cooke *et al.*, 2012; Skomal and Mandelman, 2012; Kneebone *et al.*, 2013). In the white marlin recreational fishery, the broad acceptance of circle hooks (the vast majority of which lodge in the corner of the jaw; Graves and Horodysky, 2008) has been shown to reduce greatly the variance in hooking location and the injuries caused by fishing gear. This provides an opportunity to evaluate the relative importance of physiological disturbance to post-release mortality in this important recreational fishery.

The objectives of our study were to combine a suite of physiological measurements with information from PSATs to understand better the condition and fate of white marlin captured and released in the US recreational fishery and to develop physiological predictors of post-release mortality. We hypothesized that white marlin with longer angling times would show elevated physiological stress, would have a greater likelihood of post-release mortality and would show differences in behaviour in a 4 h period following release from white marlin angled for shorter periods.

## Materials and methods

### Capture of fish and tag deployment

All fish collection and handling procedures were approved by the College of William and Mary Institutional Animal Care and Use Committee (protocol number: IACUC-2012-10-08-8165-jgraves) and followed all applicable US laws and regulations. White marlin sampling and PSAT deployments were conducted on private and charter recreational fishing vessels between August and October of 2012 and 2013. Fishing occurred 100–160 kilometers off the US mid-Atlantic coast in and around the Norfolk, Washington and Baltimore submarine canyons. Sixty-eight white marlin were captured on sportfishing tackle commonly used in the fishery: trolled ballyhoo (*Hemiramphus brasiliensis*) rigged with a 7/0 Mustad circle hook, 13.6 kg breaking strength (i.e. 30 pound test) main line, and a 27.2–45.4 kg breaking strength (60–100 pound test) leader (∼10 m in length). Angling times reflected the best effort of anglers to bring fish into the boat as quickly as possible; however, in order to manoeuvre the fish into a position close to the vessel to be safely brought on board, angling times were somewhat increased over typical recreational releases that often occur shortly after the leader can be touched. White marlin were removed from the water by hand, placed on the deck, which was wetted with seawater, and the fish’s eyes were covered with a damp towel to reduce movement and stress. We recorded lower jaw fork length (LJFL) and hook location before removal of the hook and physiological sampling.

Approximately 5 ml of blood was withdrawn from the ventral aorta using a 10 ml syringe (lithium heparin coated; BD Inc., Franklin Lakes, NJ, USA) and a 3.8 cm (1.5″) 18-gauge needle. Additionally, the first fish that was hooked in the corner of the jaw and captured in each of three angling-time categories [short (0–10 min, *n* = 8), medium (10–20 min, *n* = 7) and long (>20 min, *n* = 7)] was tagged with a High-Rate X-Tag (Microwave Telemetry Inc., Columbia, MD, USA). Angling time categories were determined *a priori* using the distribution of angling times provided by Graves and Horodysky (2008). The rigging and deployment of PSATs followed the protocol outlined by Graves *et al.* (2002). The PSATs were programmed to release from the fish after 30 days or to release if: (i) a depth of 1250 m was reached (i.e. the fish died and sank over deep-water areas, such as those to the east of the continental shelf), or (ii) depth values remained constant (±3 m) for 4 days (i.e. the PSAT was shed prematurely and was floating on the surface or the fish died and sank over waters <1250 m deep).

Individuals were returned to the water immediately after blood sampling and PSAT attachment. Once a particular angling category was filled (e.g. eight white marlin had been caught in the short angling-time category and tagged with PSATs), fish caught subsequently in the filled category were sampled for blood but not tagged and were immediately returned to the water. All fish were also resuscitated before release, a practice common in the recreational fishery. To do this, individuals were held in the water by the bill and base of the dorsal fin so that they remained underwater, oriented into the current alongside the slowly moving vessel, and oxygenated water was forced over the gills. Fish were released once they regained some normal coloration and showed signs of trying to swim. Resuscitation times ranged from ∼15 to 180 s.

The geographical location, sea surface temperature, angling time and handling time were recorded for each fish. Angling time was defined as the time from when the fish was hooked to when the fish was brought alongside the vessel, whereas handling time was defined as the time the fish was out of the water on the vessel. Our goal was to minimize handling times and keep them as consistent as possible between fish. It was not always possible to record the precise time that white marlin spent out of water, but the time on deck was recorded in 30 s intervals. Most individuals were on deck for <2 min, although handling times as long as 3.5 min were recorded for a few individuals.

### Blood sample processing

All blood samples were processed immediately after collection in the field. Haematocrit, or the proportion of blood volume composed of red blood cells, was assessed from a subsample after centrifugation at 12 000***g*** for 5 min in a microhaematocrit centrifuge (ZIPocrit; LW Scientific, Lawrenceville, GA, USA). The remaining blood sample was centrifuged (ZIPspin; LW Scientific, Lawrenceville, GA, USA) at 12 000***g*** for 5 min, the plasma decanted, immediately frozen in liquid nitrogen and, subsequently, stored at −80°C.

Plasma samples were thawed and analysed for sodium ([Na^+^]), chloride ([Cl^−^]), potassium ([K^+^]), calcium ([Ca^2+^]), magnesium ([Mg^2+^]), glucose and lactate concentrations using an automated blood chemistry analyser (NOVA CCX Statprofiler, Waltham, MA, USA). All ions and metabolites were within the detection limits of the instrument and were analysed without dilution. Plasma cortisol was assessed using a competitive enzyme-linked immunosorbent assay (ELISA; Carey and McCormick, 1998) using a Tecan GENios plate reader with Magellan software (6.55) that read absorbance at 450 nm. Plasma samples analysed for cortisol were run in duplicate and the mean values used for analysis.

### Data analysis

We calculated the minimum straight-line distance travelled for each tagged fish by determining the distance between the tagging location and the first reliable satellite contact with the floating tag (ARGOS codes 1, 2 or 3) using ArcGIS (v.10.0) and Geospatial Modeling Environment (v.0.7.2.0). We used analyses of net movements, as well as time series of light levels, water temperature and depth measurements, to assess behaviour following release and to infer mortality for tags that remained attached for at least 24 h (Horodysky and Graves, 2005). Confidence intervals for estimates of post-release mortality were derived using software developed by Goodyear (2002), which used tag data to bootstrap the proportion of inferred mortality, survival and non-reporting tags over 10 000 simulations assuming no tagging-induced or natural mortality.

Multiple linear regression was used to model physiological variables from all 68 white marlin to determine whether physiological disturbance was related to angling time, LJFL or sea surface temperature. Multiple linear regression was also used to assess whether angling time, surface water temperature, fish size and/or physiological variables from white marlin [that survived and where the PSAT remained attached for at least 24 h (*n* = 15)] could predict the median water temperature recorded by PSATs in the first 4 h following release. All physiological measurements were standardized using the expression: (x − mean(x)/standard deviation (x); to account for the difference in scale.

We examined the relationship between mortality and physiological status, angling time, LJFL and surface water temperature for all white marlin that were tagged (*n* = 22) using logistic regression models to determine predictors for post-release mortality. For these mortality models, we considered the following two scenarios: (i) a standard model that included only mortalities inferred from the tag data; and (ii) a conservative model, in which non-reporting PSATs and those that detached within 24 h of deployment were also considered mortalities. The first scenario, or standard interpretation of post-release mortality, has been frequently adopted (e.g. Heberer *et al.*, 2010) and eliminates from analyses any individual where PSAT data do not definitively indicate mortality or survival. The second scenario, or conservative interpretation of post-release mortality, has also been used (e.g. Graves *et al.*, 2002) based on the consideration that a PSAT may fail to report from a live or dead fish for multiple reasons, including mechanical failure of the release mechanism, fouling of the tag antenna by floating debris or destruction of the tag during an attempted or genuine predation event (Musyl *et al.*, 2011). Likewise, reasons for PSAT detachment within the first 24 h include human tagging error and attempted or real predation of the fish that dislodges the tag (Musyl *et al.*, 2011). Shark predation of fish carrying PSATs has been documented (e.g. Kerstetter *et al.*, 2004) and is one of several possibilities why one PSAT failed to report during our study and two other tags detached within the first 24 h. We include a conservative interpretation of mortality because eliminating non-reporting tags and tags that detach too early to indicate survival decreases the precision of the estimate of post-release mortality and may artificially lower estimates of post-release mortality.

For each regression analysis, several model parameterizations were considered, and Akaike’s information criterion (AIC; Akaike, 1973; Burnham and Anderson, 2002) was used to discriminate among competing formulations. Collinearity among predictor variables was evaluated by calculating variance inflation factors. For the post-release mortality analysis, values of the predictor variables that corresponded to the 0.50 probability of mortality were calculated from the most supported model and applied to white marlin that had blood drawn but were not tagged (*n* = 46) to determine what percentage of untagged white marlin had a >0.50 probability of mortality. Student’s unpaired *t*-test was used to compare the angling times for post-release mortalities and survivors under both assumptions of mortality. All statistical analyses were conducted in R (version 3.1.0; R Core Team, 2014).

## Results

### Capture of fish and tag deployment

White marlin ranged in size from 122 to 175 cm LJFL (mean = 156 cm LJFL, standard deviation (sd) = 9 cm). Angling times were 3–41 min (mean = 14 min, sd = 9 min), encompassing extremes for the fishery (Horodysky and Graves, 2005). Surface water temperatures in capture locations ranged from 23.1 to 27.5°C (mean = 25.0°C, sd = 1.1°C). Air exposure times ranged from 90 to 210 s (mean = 120s, sd = 40 s).

All but one of the 22 PSATs deployed reported data, although four of the reporting tags detached prematurely (Table 1). Two of the PSATs that detached early released 11 and 12 days post-deployment, but both of these PSATs recorded temperature, depth and horizontal movement data that demonstrated survival during the time the tags were attached and were included as survivors in the post-release mortality analysis. The remaining two premature releases detached <24 h after deployment, a period we considered too short to assume survival. Therefore, the two tags that detached within 24 h and the one non-reporting tag were eliminated from the standard estimate of post-release mortality (19 PSATs) and treated as mortalities in the second, more conservative estimate of post-release mortality (22 PSATs).

**Table 1:** Lower jaw fork length, angling time, air exposure time (measured in 30 s intervals), tag duration (time for which the tag remained attached to the fish), straight-line distance travelled and fate of 22 white marlin caught on circle hooks in the US recreational fishery and tagged with 30 day pop-up satellite archival tags

LJFL (cm)	Angling time (min)	Air exposure ’(min; 30 s intervals)	Tag duration (days)	Straight-line distance ’travelled (km)	Fate
147	17	2	30	1002	S
147	14	3.5	30	2086	S
173	8	2	30	449	S
155	10	2	30	1268	S
160	33	2	30	1779	S
160	8	3.5	<1	–	24 h
155	12	2	12	666	S
155	20	2	30	1951	S
155	6	3.5	30	539	S
164	36	2	30	1119	S
154	41	1.5	30	2643	S
160	28	3	11	242	S
165	8	2	4	–	M
175	6.5	3	30	91	S
163	18.5	2.5	30	737	S
137	16	3.5	4	–	M
163	21	2	–	–	NR
154	24	2	30	799	S
165	10	2	4	–	M
168	21	2	<1	–	24 h
165	5	2	4	–	M
160	26	2	30	888	S

Abbreviations: LJFL, lower jaw fork length; M, mortality; NR, non-reporting; S, survivor; and 24 h, release within 24 h.

Four unambiguous post-release mortality events were also confirmed from PSAT data. These four mortality events all occurred immediately after release and were marked by fish sinking slowly to the sea floor. In our standard interpretation of post-release mortality, four mortality events were inferred from 19 tags for a post-release mortality probability of 0.21 [95% confidence interval 0.05–0.37, based on the bootstrap method of Goodyear (2002)]. In our conservative interpretation of post-release mortality, seven post-release mortality events were inferred from 22 tags, for a post-release mortality probability of 0.32 [95% confidence interval 0.14–0.46, based on the bootstrap method of Goodyear (2002)]. For both the standard and conservative assumptions of mortality, angling times were significantly shorter for white marlin that died post-release than for those that survived (*t*-test, *P* < 0.01 and *P* < 0.05, respectively).

### Physiological samples

**Figure 1:**
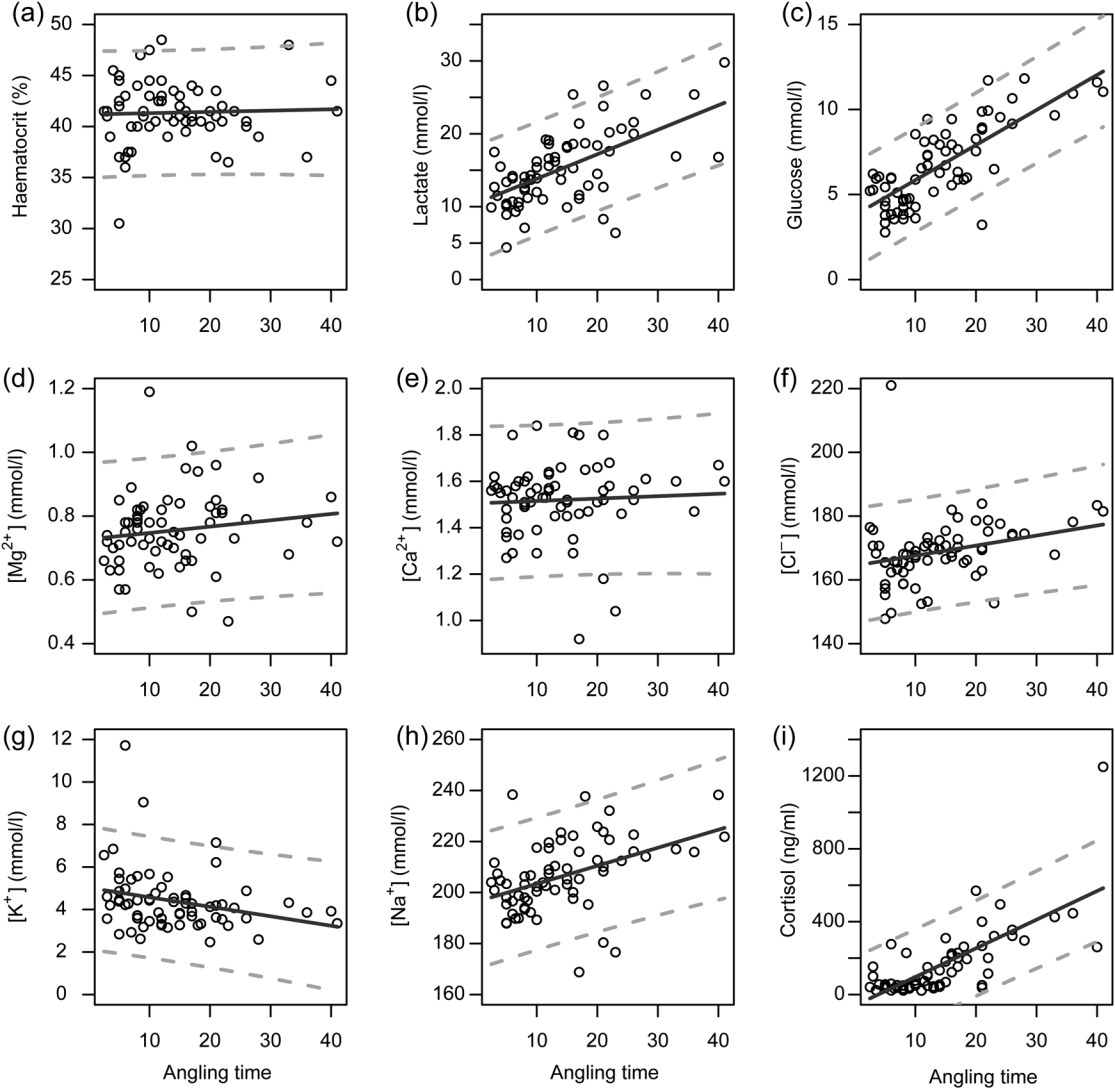
(**a**–**i**) Model fit for linear regressions fitted to physiological data from blood samples collected from 68 white marlin caught in the US recreational fishery. Continuous line shows model predictions and dashed lines show 95% prediction intervals.

Regression models that used only angling time to predict haematocrit, cortisol, [Mg^2+^], [Ca^2+^] and [Na^+^] received the most empirical support based on AIC, although other parameterizations received competing support (ΔAIC ≤ 4; Table 2). Haematocrit, cortisol, [Mg^2+^], [Ca^2+^] and [Na^+^] all increased with angling time (Table 3 and Fig. 1a, d, e, h and i). Regression models that used angling time and sea surface temperature received the most empirical support to predict lactate, glucose, [Cl^−^] and [K^+^], and again, other model formulations provided competing descriptions of the data (Table 2). Concentrations of lactate, glucose and [Cl^−^] increased with both angling time and sea surface temperature, whereas [K^+^] increased with sea surface temperature but decreased with angling time (Table 3 and Fig. 1b, c, f and g).

**Table 2:** Model fit and comparison statistics for linear regressions fitted to physiological data from 68 white marlin caught in the US recreational fishery

Response variable	Explanatory variable(s)	Number of parameters	Negative log likelihood	AIC	ΔAIC
Haematocrit (%)	AT + SST + LJFL	5	168.48	346.96	3.99
	AT + SST	4	168.48	344.97	2.00
	AT + LJFL	4	168.48	344.97	2.00
	AT	3	168.49	342.97	0.00
Lactate (mmol/l)	AT + LJFL + SST	5	183.33	376.66	0.38
	AT + SST	4	184.14	376.28	0.00
	AT + LJFL	4	187.53	383.07	6.78
	AT	3	188.26	382.52	6.24
Glucose (mmol/l)	AT + LJFL + SST	5	121.28	252.56	1.30
	AT + SST	4	121.63	251.26	0.00
	AT + LJFL	4	122.41	252.83	1.57
	AT	3	122.75	251.51	0.25
[Mg^2+^] (mmol/l)	AT + LJFL + SST	5	−50.25	−90.50	3.53
	AT + SST	4	−50.15	−92.29	1.74
	AT + LJFL	4	−50.13	−92.25	1.79
	AT	3	−50.02	−94.04	0.00
[Ca^2+^] (mmol/l)	AT + LJFL + SST	5	−28.63	−47.27	2.51
	AT + SST	4	−28.61	−49.22	0.59
	AT + LJFL	4	−27.91	−47.83	1.96
	AT	3	−27.89	−49.78	0.00
[Cl^−^] (mmol/l)	AT + LJFL + SST	5	238.69	487.38	1.37
	AT + SST	4	239.00	486.00	0.00
	AT + LJFL	4	246.97	501.94	15.94
	AT	3	247.22	500.44	14.45
[K^+^] (mmol/l)	AT + LJFL + SST	5	116.76	243.51	1.81
	AT + SST	4	116.85	241.70	0.00
	AT + LJFL	4	117.83	243.65	1.96
	AT	3	117.92	241.84	0.14
[Na^+^] (mmol/l)	AT + LJFL + SST	5	264.97	539.94	3.52
	AT + SST	4	264.97	537.94	1.52
	AT + LJFL	4	265.21	538.42	2.00
	AT	3	265.21	536.42	0.00
Cortisol (ng/ml)	AT + LJFL + SST	5	419.94	849.88	3.63
	AT + SST	4	420.11	848.22	1.98
	AT + LJFL	4	419.95	847.90	1.66
	AT	3	420.12	846.24	0.00

Abbreviations: AIC, Akaike’s information criterion; AT, angling time; LJFL, lower jaw fork length; and SST, sea surface temperature.

**Table 3:** Model summaries from the linear regressions that received the most empirical support for each measured physiological variable derived from 68 white marlin caught in the US recreational fishery

Response variable	Explanatory variable(s)	Parameter estimates	Standard error	*t*-value	*P*-value
Haematocrit (%)	AT	0.013	0.042	0.30	0.77
Lactate (mmol/l)	AT	0.336	0.054	6.22	<0.001
	SST	0.711	0.245	2.90	<0.01
Glucose (mmol/l)	AT	0.206	0.021	9.70	<0.001
	SST	0.142	0.097	1.48	>0.05
[Mg^2+^] (mmol/l)	AT	0.002	0.002	1.22	0.23
[Ca^2+^] (mmol/l)	AT	0.001	0.002	0.46	0.65
[Cl^−^] (mmol/l)	AT	0.315	0.123	2.57	<0.05
	SST	2.352	0.557	4.22	<0.001
[K^+^] (mmol/l)	AT	−0.045	0.020	−2.26	<0.05
	SST	0.130	0.090	1.44	0.15
[Na^+^] (mmol/l)	AT	0.707	0.179	3.95	<0.001
Cortisol (ng/ml)	AT	15.719	1.814	8.67	<0.001

Abbreviations: AT, angling time; and SST, sea surface temperature.

The most supported multiple linear regression model to predict median temperature occupied by white marlin in the first 4 h after release used LJFL and [Ca^2+^], although neither parameter was statistically significant (Table 4). The median 4 h water temperature increased (e.g. fish spent time at shallower depths) in conjunction with increased LJFL, and decreased (e.g. fish spent time at greater depths) with increased [Ca^2+^] (for LJFL, estimate = 0.137, standard error = 0.082, *t* = 1.67, *P* = 0.12; and for [Ca^2+^], estimate = −2.736, standard error = 1.287, *t* = −2.13, *P* = 0.06).

**Table 4:** Model fit and comparison statistics for linear regressions fitted to median water temperature occupied during the first 4 h following release (initial habitat utilization) of 15 white marlin tagged with pop-up satellite archival tags

Response variable	Explanatory variable(s) and coefficient	Number of parameters	Negative log likelihood	AIC	ΔAIC
Initial habitat utilization	LJFL + GLU + COR + [K^+^] + [Ca^2+^] (+, +, +, +, −)	7	17.88	49.76	5.18
	LJFL + GLU + COR + [Ca^2+^] (+, +, +, −)	6	18.04	48.07	3.49
	GLU + [K^+^] + [Ca^2+^] (−, +, −)	5	19.73	49.47	4.89
	LJFL + [K^+^] + [Ca^2+^] (+, +, −)	5	18.23	46.47	1.89
	[Ca^2+^] + GLU + LJFL (−, +, +)	5	18.17	46.35	1.77
	COR + [Ca^2+^] (+, −)	4	19.85	47.70	3.12
	LJFL + [Ca^2+^] (+, −)	4	18.29	44.58	0.00
	[Ca^2+^] + GLU (−, −)	4	19.82	47.64	3.07
	COR (−)	3	21.18	48.36	3.79
	GLU (−)	3	20.86	47.73	3.15
	[Ca^2+^] (−)	3	19.86	45.71	1.13
	[K^+^] (+)	3	20.82	47.64	3.07

Abbreviations: AIC, Akaike’s information criterion; COR, plasma cortisol concentration; GLU, plasma glucose concentration; and LJFL, lower jaw fork length.

**Figure 2:**
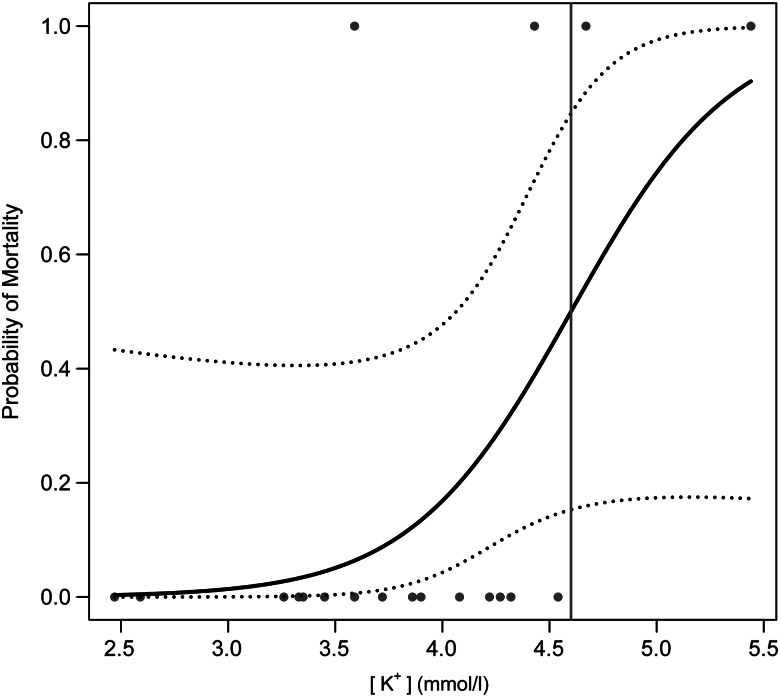
Predicted probability of post-release mortality from the most supported logistic regression model fitted to the post-release pop-up satellite archival tag data for white marlin caught in the US recreational fishery under the standard interpretation of non-reporting and detached tags (15 survivors and four mortalities). Dotted lines show 95% confidence intervals, and the vertical line denotes the 0.50 probability of mortality at 4.60 mmol/l [K^+^].

**Figure 3:**
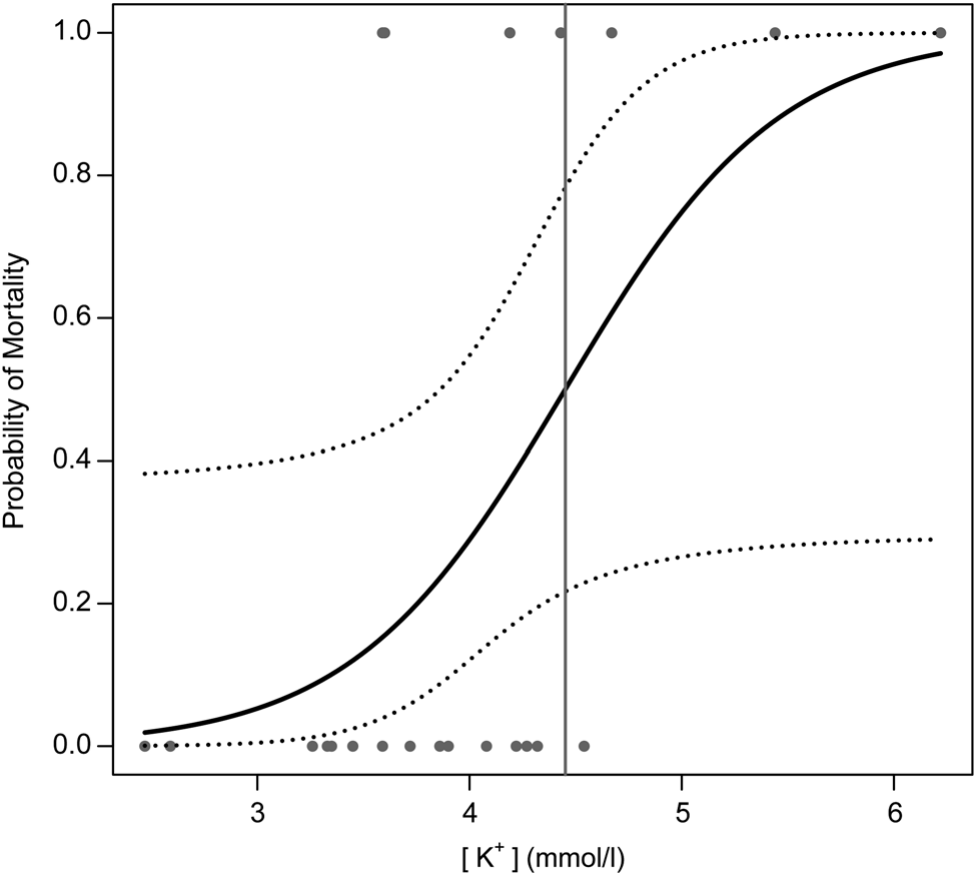
Predicted probability of post-release mortality from the most supported logistic regression model fitted to the post-release pop-up satellite archival tag data for white marlin caught in the US recreational fishery under the conservative interpretation of non-reporting and detached tags (15 survivors and seven mortalities). Underlying data included four mortalities shown in Fig. 2 plus one non-reporting tag and two tags that detached within 24 h of being deployed that were assumed to be mortalities. Dotted lines show 95% confidence intervals, and the vertical line denotes the 0.50 probability of mortality at 4.45 mmol/l [K^+^].

Logistic regression models using only [K^+^] received the most empirical support for both the standard and the conservative interpretations of post-release mortality, although other model structures received competing support (Table 5). The probability of mortality increased with [K^+^], but the standard errors of the estimated coefficients of [K^+^] were relatively large and likely to be due to the low mortality in both data sets (in the standard mortality model, estimate = 2.66, standard error = 1.51, *Z* = 1.76, *P* = 0.08; and in the conservative mortality model, estimate = 1.99, standard error = 1.03, *Z* = 1.93, *P* = 0.05). The estimate for the standard logistic regression model that used four mortalities established the probability of 0.50 mortality at [K^+^] = 4.60 mmol/l (Fig. 2), placing 17 of the 46 (37%) white marlin that were sampled but not tagged above the 0.50 probability of mortality. The estimate for the conservative logistic regression model used seven mortalities and established the probability of 0.50 mortality at [K^+^] = 4.45 mmol/l (Fig. 3), placing 21 of the 46 (46%) white marlin that were sampled but not tagged above the 0.50 probability of mortality. Both of these mortality estimates fall at the upper limit of the 95% confidence intervals derived solely from PSAT data that were estimated using the software from Goodyear (2002).

**Table 5:** Model fit and comparison statistics for logistic regressions fitted to post-release mortality data for white marlin caught in the US recreational fishery

Response variable	Explanatory variable(s) and coefficient	Number of parameters	Negative log likelihood	AIC	ΔAIC
Standard	[Na^+^] (−)	2	9.66	23.33	5.58
post-release	COR (−)	2	8.29	20.58	2.83
mortality	[K^+^] (+)	2	6.87	17.75	0.00
	[Mg^2+^] (+)	2	9.42	22.85	5.10
	HCT (+)	2	9.78	23.56	5.81
	[Cl^−^] (+)	2	9.76	23.52	5.77
	AT (−)	2	7.74	19.48	1.73
	[Mg^2+^] + AT (+, −)	3	7.50	21.01	3.26
	[K^+^] + AT (+, −)	3	6.07	18.13	0.39
	[K^+^] + AT + SST(+, −, +)	4	6.01	20.03	2.28
	[K^+^] + AT + SST + LJFL (+, −, +, −)	5	5.90	21.80	4.05
Conservative	[Na^+^] (−)	2	13.73	31.46	6.92
post-release	COR (−)	2	12.31	28.61	4.08
mortality	[K^+^] (+)	2	10.27	24.54	0.00
	[Mg^2+^] (+)	2	13.05	30.11	5.57
	HCT (−)	2	13.76	31.52	6.98
	[Cl^−^] (+)	2	13.73	31.46	6.92
	AT (−)	2	12.33	28.67	4.13
	[Mg^2+^] + AT (+, −)	3	11.68	29.35	4.82
	[K^+^] + AT (+, −)	3	9.34	24.68	0.15
	[K^+^] + AT + SST (+, −, −)	4	9.28	26.56	2.02
	[K^+^] + AT + SST + LJFL (+, −, −, +)	5	9.24	28.48	3.95

Models were applied to data both for a standard interpretation of post-release mortality where a non-reporting PSAT and two PSATs that detached within 24 h were not included (15 survivors and four mortalities) and for a conservative approach where the non-reporting PSAT and the two PSATs that detached within 24 h were assumed to be mortalities (15 survivors and seven mortalities). Abbreviations: AIC, Akaike’s information criterion; AT, angling time; COR, plasma cortisol concentration; GLU, plasma glucose concentration; HCT, haematocrit; LAC, plasma lactate concentration; LJFL, lower jaw fork length; PSAT, pop-up satellite archival tag; and SST, sea surface temperature.

## Discussion

Our objectives were to combine a suite of physiological measurements with information from PSATs to gain a better understanding of the condition and fate of white marlin captured and released in the US recreational fishery and to develop physiological predictors of post-release mortality. Despite a relatively small sample size, our data show that physiological stress has a direct link to post-release mortality. Elevated [K^+^] proved to be the sole predictor of post-release mortality regardless of whether a standard or conservative mortality scenario was considered. Several previous studies have also noted elevated [K^+^] in response to stress events in teleost and elasmobranch fishes (Cliff and Thurman, 1984; Wells *et al.*, 1986; Manire *et al.*, 2001; Mandelman and Farrington, 2007; Frick *et al.*, 2010; Danylchuk *et al.*, 2014) and correlated elevated [K^+^] with rates of post-release mortality in some species of carcharhinid sharks (Moyes *et al.*, 2006; Marshall *et al.*, 2012). Furthermore, elevated [K^+^] has been shown to be a significant predictor for mortality in the dusky shark (*Carcharhinus obscurus*; personal communication, H. Marshall and D. Bernal, University of Massachusetts, Dartmouth, MA, USA), although [K^+^] thresholds for predicting post-release mortality were significantly higher in this species than in white marlin. Potassium is largely an intracellular ion, and the presence of elevated [K^+^] (i.e. hyperkalaemia) may result from cellular damage releasing intracellular contents and/or acidosis. The latter can itself induce cellular damage, modify electrochemical gradients and affect locomotor and heart muscle function (Cliff and Thurman, 1984; Moyes *et al.*, 2006).

Our models also suggest that physiological stress may influence behaviour and habitat utilization following release. Smaller white marlin with elevated [Ca^2+^] spent a greater proportion of time in cooler water in the first 4 h following release than those with lower levels of [Ca^2+^]. Elevations in [Ca^2+^] and catecholamine levels have been proposed as a protective cardiac mechanism from stress-induced acidosis in teleosts (Farrell, 1984). Furthermore, elevated [Ca^2+^] has been observed previously in recreationally angled istiophorids (Wells *et al.*, 1986) and identified as a discriminating factor between moribund and surviving blue sharks (Moyes *et al.*, 2006). Researchers have previously hypothesized that blue marlin seek cooler temperatures for 4–6 h following recreational angling as a physiological response to severe anaerobic debt associated with capture (Holland *et al.*, 1990a; Block *et al.*, 1992), and our data are consistent with this hypothesis.

In addition to the stress and trauma associated with catch and release, the process of tagging fish with PSATs has itself been hypothesized to affect behaviour. A recent meta-analysis of 183 large pelagic fishes tagged with PSATs suggested that 37% of all individuals, and 20% of white marlin, exhibited irregular behaviour from 3 to 60 days following release, which the authors attributed to the stress of capture as well as the attachment of the PSAT (Hoolihan *et al.*, 2011). Comparison of vertical movement behaviours from acoustic telemetry studies (conducted for ∼24–48 h following release) and studies using implanted archival tags and PSATs that often contain months-long data records (e.g. Carey and Robison, 1981; Holland *et al.*, 1990b; Brill *et al.*, 1993; Block *et al.*, 1997; Dewar *et al.*, 2011; Schaefer *et al.*, 2011; Chiang *et al.*, 2015; Lam *et al.*, 2015) show remarkable similarity and suggest that: (i) recovery following release in tunas and billfishes requires only hours; and (ii) changes in vertical movement patterns over longer periods of time can be explained largely by differences in oceanographic conditions and prey distributions. The influence of stress on the behaviour of large, PSAT-tagged, pelagic fishes following release merits further research given the breadth of information suggesting behaviour modification and our analyses that suggest a relationship between fish size, [Ca^2+^] and water temperature used in the initial 4 h period after release.

With the exception of [K^+^], angling time increased measures of physiological stress in white marlin. The increases in plasma lactate, [Cl^−^], [Na^+^], cortisol and glucose that we observed with angling time in white marlin are similar in magnitude to those reported for white marlin by Skomal (2006) and for other fishes, including rainbow trout (*Oncorhynchus mykiss*; Wood *et al.*, 1983), bonefish (*Albula vulpes*; Suski *et al.*, 2007), Caribbean reef sharks (*Carcharhinus perezi*; Brooks *et al.*, 2012) and a variety of inshore and pelagic sharks (Marshall *et al*., 2012). However, without repeated sampling of individual fish it is impossible to discern whether elevations in physiological parameters that occur with increased angling time reflect a greater degree of stress or the natural time course of the physiological response to stress (Skomal and Bernal, 2010). Plasma lactate, [K^+^] and [Cl^−^] also increased appreciably at higher sea surface temperatures in white marlin. Likewise, the effect of increased water temperature has been found to amplify physiological stress in largemouth bass (*Micropterus salmoides*; Suski *et al.*, 2006).

**Figure 4:**
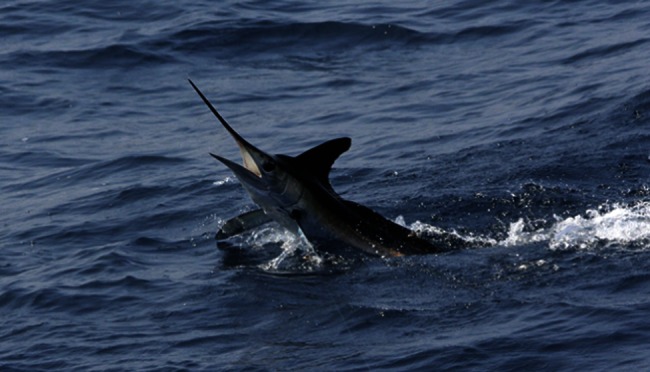
A white marlin thrashes at the surface during a typical catch-and-release event. White marlin often jump and surface repeatedly during angling events, which can make even short angling times stressful. Photograph by Ken Neill, Healthy Grin Sportfishing.

Despite the prevalence of angling time in our physiological models, longer angling time was not a predictor for post-release mortality or behaviour following release. However, in contrast to our initial hypotheses and those of other authors (e.g. Musyl *et al.*, 2015), angling times were significantly shorter for fish that died post-release than for those that survived, indicating that in cases where fish behaviour can vary greatly during angling, the variable angling time may not be an appropriate proxy for stress. Likewise, post-release mortality of recreationally angled shortfin mako (*Isurus oxyrinchus*), a pelagic elasmobranch with a high aerobic scope, occurred only for fish angled <30 min (French *et al.*, 2015). In the white marlin fishery, angling times are typically short when the fish jumps frequently and pulls line off the reel against high drag, which has the effect of exhausting the fish quickly. In contrast, longer angling events are often marked by a fish that swims to depth and uses caudal propulsion as well as its surface area in combination with water currents to provide resistance. We suggest that short, intense angling events may represent a ‘sprint’ for white marlin (i.e. involve a high proportion of anaerobic muscle activity), whereas longer angling events are a ‘jog’ (i.e. involve a high proportion of aerobic muscle activity). This hypothesis is further corroborated by the significant inverse relationship we observed between [K^+^], a predictor for mortality, and angling time. Istiophorid billfishes have a high white muscle buffering capacity (Abe *et al.*, 1985; Dobson *et al.*, 1986, Dickson, 1995), which allows these fish rapidly to generate high levels of white muscle lactate, and short angling events involving intense activity and anaerobic metabolism in the white muscle are likely to result in a much greater oxygen debt than longer angling events that primarily involve aerobic metabolism (Fig. 4).

Estimates of probabilities of white marlin post-release mortality inferred from our PSAT data were either 0.21 or 0.32 depending on whether we included only those tags that clearly indicated mortality (four of 19) or also included non-reporting tags and tags that detached within 24 h of deployment as mortalities (seven of 22), but in either case our estimates were more than 10 times greater than the probability of post-release mortality previously estimated for white marlin (<0.02; two of 59) by Graves and Horodysky (2008). Our models using [K^+^] as a predictor of post-release mortality additionally placed 37 or 46% of the white marlin that we sampled (but that were not tagged) above the 0.50 probability of mortality, further suggesting the possibility of elevated post-release mortality levels. It is important to consider that our results are based on relatively few mortalities, with 95% confidence intervals ranging from 0.05 to 0.37 and from 0.14 to 0.46 for the standard and conservative mortality models, respectively, and should therefore be applied cautiously. Angling times we measured (range = 3–41 min, $\bar{t}=14\;\;\min$) were not significantly different from those recorded by Graves and Horodysky 2008, (range = 4–40 min, $\bar{t}=13\;\;\min$), and in both studies the same type of gear and the same style of fishing were used. In addition, the PSATs were deployed in a similar manner, and fish were resuscitated prior to release in both studies. However, unlike Graves and Horodysky (2008), we removed fish from the water for about 2 min to allow blood sampling. We therefore suggest that the large disparity in mortality estimates between the two studies was a result of the brief air exposure that fish experienced prior to release.

Air exposure has been shown to affect behaviour and to increase physiological stress and post-release mortality in fishes (Danylchuk et al., 2007; Gingerich *et al.*, 2007; Cook *et al.*, 2015). Time out of water reduces effective oxygen uptake and carbon dioxide offloading at the gills. This, in turn, results in respiratory acidosis (Ferguson and Tufts, 1992), an increase in blood lactate levels (Arends *et al*., 1999; Davis and Schreck, 2005; Suski *et al.*, 2007), cardiovascular alterations (Cooke *et al.*, 2003) and osmotic/ionic disruptions (Waring *et al.*, 1996; Milston *et al.*, 2006; Hur *et al.*, 2007). Air exposure in combination with exercise is likely to be especially stressful in white marlin when a large oxygen debt has been accumulated as a result of a short, intense angling event when anaerobic burst swimming is predominant. Bonefish exercised to exhaustion and then exposed to air required more than 10 times as long to regain equilibrium and had blood lactate nearly three times greater initially, and nearly twice as high after a 2 h recovery, than bonefish exposed to air but not exercised (Suski *et al*., 2007). Likewise, bonefish handled and exposed to air after recreational capture were found to have difficulty maintaining equilibrium (Cooke and Philipp, 2004), and individuals that lost equilibrium were six times more likely to suffer predation (Danylchuk *et al.*, 2007). During blood sampling, we did our best to ensure that air exposure was brief and consistent between fish. We therefore hypothesize that air exposure had much greater consequences for fish that had exerted themselves anaerobically during a short angling event, and thus had a greater oxygen debt, than for those that exhibited aerobic swimming during a longer angling event.

Handling and blood sampling are also likely to increase physiological stress and are recognized as an inherent problem in physiological stress studies (Cooke *et al.*, 2012). Despite the importance of considering the added stress of sampling, we find it unlikely that collecting such a small fraction of total blood volume in itself caused the differences in probabilities of post-release mortality between the present study and the results of Graves and Horodysky (2008). Additionally, the elevation of [K^+^] we measured could not have been the result of sampling and presumably reflects individual differences in the level of exertion during angling.

A recent meta-analysis of published PSAT data by Musyl *et al.* (2015) reported that survival of released istiophorid billfishes tends to be high (∼86%) across different species, fisheries and terminal tackle. However, owing to limited sample sizes, few studies had sufficient statistical power to evaluate the effect of many variables. To date, only differences in hook type (Horodysky and Graves, 2005; Graves and Horodysky, 2008) and release location (Horodysky and Graves, 2005; Prince *et al*., 2005) have been evaluated with relatively robust sample sizes. Our results indicate that handling procedures (primarily air exposure associated with blood sampling) also result in significant differences in probabilities of post-release mortality.

Our results demonstrate the importance of evaluating different variables when estimating post-release mortality, particularly in a fishery with a large number of recreational fishers across many countries that have different styles of catching, handling and releasing fish. Additionally, this study illustrates the importance of pairing physiological sampling with the use of PSATs to gain a better understanding of the relationship between catch-and-release fishing practices, physiological stress, mortality and behaviour following release. We are the first to evaluate the physiological responses of white marlin to catch-and-release angling with a sample size sufficiently large for statistical inferences, and we suggest that relationships exist between physiological stress and the probability of mortality and between physiological stress and behaviour in the 4 h period following release. The relationship between elevated [K^+^] and mortality, a response similarly observed in several carcharhinid sharks (Moyes *et al.*, 2006; Marshall *et al.*, 2012), suggests a commonality across fish species.

**Figure 5:**
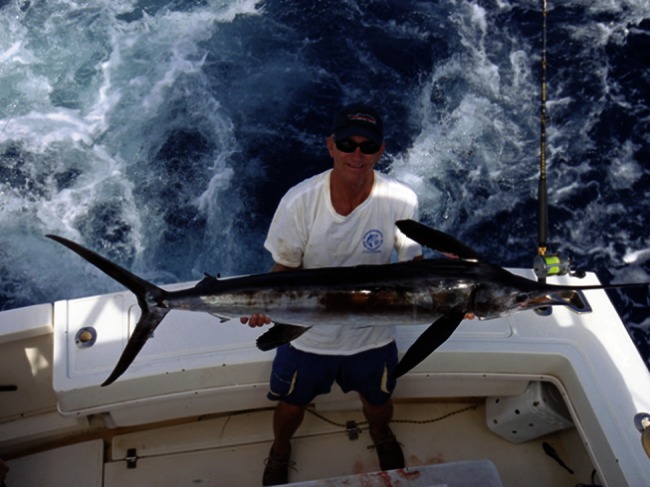
A recreational angler holds a white marlin out of water for a photograph. Despite the fact that removing white marlin from the water is illegal in the USA, many anglers bring white marlin into the boat to take pictures before releasing them. Even a relatively short period out of water induces additional stress from air exposure and handling, which may increase the probability of post-release mortality. Photograph by Ken Neill, Healthy Grin Sportfishing.

Understanding the effects of the stress associated with capture and release is crucial to conservation and management practices and policies. Our results suggest that the probability of post-release mortality may be greatly affected by air exposure immediately after capture. Removal of white marlin from the water is illegal in the USA, but many recreational anglers are unaware of this law and bring fish into the boat for pictures (Fig. 5). Effective outreach is necessary to demonstrate to anglers that post-release survival of white marlin is crucially dependent on post-capture handling. It is our hope that being able to provide evidence to the offshore angling community that removal of fish from the water significantly increases post-release mortality will increase compliance with current regulations and that this will ultimately benefit the survival and conservation of this highly sought-after and economically valuable species.

## Funding

This work was supported by the Offield Family Foundation and the National Science Foundation GK-12 (DGE - 0840804 to L.S.S.).
